# Supplementary material for: Zero-inflated models for the evaluation of colorectal polyps in colon cancer screening studies—a value-based biostatistics practice
Source: PeerJ. 2025 May 26;13:e19504. doi: 10.7717/peerj.19504 (PMC12121622; doi:10.7717/peerj.19504)
Supplement: Supplemental Information 1 [file peerj-13-19504-s001.docx]

Supplementary Table 1. Baseline characteristics of patients in each dataset

| **Factor** | **Dataset1** | **Dataset2** | **Dataset3** |
| --- | --- | --- | --- |
| **N** | **425** | **311** | **1936** |
| Age, mean (SD) | 59.76 (7.19) (n=425) | 57.88 (6.60) (n=311) | 58.72 (6.58) (n=1936) |
| Sex |  |  |  |
| Female | 295 (69.4%) | 203 (65.3%) | 1284 (66.3%) |
| Male | 130 (30.6%) | 108 (34.7%) | 652 (33.7%) |
| BMI, mean (SD) | 30.14 (6.13) (n=425) | 30.96 (5.70) (n=311) | 30.62 (5.82) (n=1936) |
| Preparation_Quality |  |  |  |
| Excellent | 49 (11.5%) | 17 (5.5%) | 393 (20.3%) |
| Good | 273 (64.2%) | 219 (70.4%) | 1308 (67.6%) |
| Fair | 103 (24.2%) | 67 (21.5%) | 150 (7.7%) |
| Poor | 0(0%) | 8 (2.6%) | 85 (4.4%) |
| Procedure_time, mean (SD) | 19.85 (9.61) (n=425) | 26.41 (11.29) (n=309) | 25.83 (14.90) (n=1117) |

SD: standard devaition; BMI: body mass index

Supplementary Figure 1. Distribution of polyp count in each dataset

Supplementary Table 2. Sensitivity analysis for comparison of polyp detection rate and the number of detected polyps between groups without adjusting for covariates in clinical trials, and adjusting for all baseline variables in the analysis of dataset 3

| **Models** | **Polyp detection rate** | | **Number of detected polyps** | |
| --- | --- | --- | --- | --- |
|  | **OR (95% CI)** | **p-value** | **RR (95% CI)** | **p-value** |
| **Example dataset 1: CAC vs. SC procedures** | | | | |
| LR | 0.93 (0.63, 1.37) | 0.714 | *NA* | |
| PR | *NA* | | 1.21 (0.99, 1.49) | 0.068 |
| RP | *NA* | | 1.21 (0.90, 1.63) | 0.203 |
| NB | *NA* | | 1.21 (0.89, 1.65) | 0.220 |
| ZIP | 0.64 (0.36, 1.13) | 0.120 | 1.49 (1.13, 1.97) | **0.004** |
| ZIRP | 0.64 (0.34, 1.18) | 0.149 | 1.49 (1.06, 2.11) | **0.023** |
| ZINB^#^ | 0.55 (0.25, 1.22) | 0.142 | 1.44 (1.09, 1.91) | **0.011** |
| ZIGP^#^ | 0.53 (0.23, 1.25) | 0.146 | 1.45 (1.09, 1.93) | **0.011** |
| ZHP | 0.93 (0.63, 1.37) | 0.714 | 1.49 (1.13, 1.97) | **0.004** |
| ZHNB | 0.93 (0.63, 1.37) | 0.714 | 1.60 (1.06, 2.41) | **0.025** |
| **Example dataset 2: Segmental vs. non-segmental withdrawal time protocols** | | | | |
| LR | 1.42 (0.91, 2.22) | 0.122 | *NA* | |
| PR | *NA* | | 1.17 (0.95, 1.44) | 0.141 |
| RP | *NA* | | 1.17 (0.88, 1.56) | 0.289 |
| NB | *NA* | | 1.17 (0.86, 1.58) | 0.311 |
| ZIP | 1.59 (0.85, 2.98) | 0.146 | 0.98 (0.76, 1.27) | 0.900 |
| ZIRP | 1.59 (0.83, 3.05) | 0.160 | 0.98 (0.73, 1.33) | 0.915 |
| ZINB | 2.16 (0.55, 8.49) | 0.269 | 0.98 (0.69, 1.39) | 0.918 |
| ZIGP | 2.40 (0.51, 11.29) | 0.267 | 0.92 (0.61, 1.39) | 0.689 |
| ZHP | 1.42 (0.91, 2.22) | 0.122 | 0.98 (0.76, 1.27) | 0.900 |
| ZHNB | 1.42 (0.91, 2.22) | 0.122 | 0.98 (0.69, 1.39) | 0.918 |
| **Example dataset 3: After the GI fellowship program vs. before the GI fellowship program*** | | | | |
| LR | 1.64 (1.36, 1.97) | **<0.001** | *NA* | |
| PR | *NA* | | 1.57 (1.42, 1.74) | **<0.001** |
| RP | *NA* | | 1.57 (1.37, 1.80) | **<0.001** |
| NB | *NA* | | 1.57 (1.36, 1.80) | **<0.001** |
| ZIP | 1.41 (1.04, 1.90) | **0.026** | 1.35 (1.17, 1.56) | **<0.001** |
| ZIRP | 1.41 (1.02, 1.94) | **0.039** | 1.35 (1.14, 1.61) | **0.001** |
| ZINB | 2.24 (0.64, 7.88) | 0.208 | 1.49 (1.17, 1.90) | **0.001** |
| ZIGP | 2.65 (0.79, 8.85) | 0.114 | 1.29 (1.02, 1.65) | **0.036** |
| ZHP | 1.64 (1.36, 1.97) | **<0.001** | 1.36 (1.18, 1.57) | **0.001** |
| ZHNB | 1.64 (1.36, 1.97) | **<0.001** | 1.44 (1.19, 1.76) | **0.036** |

OR: Odds Ratio; RR: Risk Ratio; CI: Confidence Interval; NA: Not Applicable; LR: Logistic Regression; PR: Poisson Regression; RP: Robust Poisson; NB: Negative Binomial; ZIP: Zero-inflated Poisson; ZIRP: Zero-inflated Robust Poisson; ZINB: Zero-inflated Negative Binomial; ZIGP: Zero-inflated Generalized Poisson; ZHP: Zero hurdle Poisson; ZHNB: Zero hurdle Negative Binomial; CAC: Cap-Assisted Colonoscopy; SC: Standard Colonoscopy; GI: Gastroenterology; ^#^Adjusted for total procedure time; *Adjusted for age, sex, body mass index, time of day, and sedation type; Highlighted p-values are significant findings.

Supplementary Table 3. Comparison of the polyp detection rate and number of detected polyps between groups on all polyp counts

| **Models** | **Polyp detection rate** | | **Number of detected polyps** | |
| --- | --- | --- | --- | --- |
|  | **OR (95% CI)** | **p-value** | **RR (95% CI)** | **p-value** |
| **Example dataset 1: CAC vs. SC procedures#** | | | | |
| LR | 0.92(0.61,1.39) | 0.702 | *NA* | |
| PR | *NA* | | 1.21(0.98,1.48) | 0.071 |
| RP | *NA* | | 1.21(0.92,1.57) | 0.17 |
| NB | *NA* | | 1.20(0.91,1.57) | 0.198 |
| ZIP | 0.56(0.89,1.17) | 0.120 | 1.44(1.12,1.87) | **0.005** |
| ZIRP | 0.56(0.87,1.22) | 0.143 | 1.44(1.05,1.99) | **0.024** |
| ZINB | 0.48(0.85,1.34) | 0.163 | 1.45(1.06,1.98) | **0.019** |
| ZIGP | 0.42(0.86,1.38) | 0.153 | 1.49(1.09,2.03) | **0.012** |
| ZHP | 0.92(0.61,1.39) | 0.702 | 1.47(1.05,2.04) | **0.024** |
| ZHNB | 0.92(0.61,1.39) | 0.702 | 1.53(1.10,2.13) | **0.012** |
| **Example dataset 2: Segmental vs. non-segmental withdrawal time protocols#** | | | | |
| LR | 1.64(1.01,2.67) | **0.045** |  | |
| PR | *NA* | | 1.09(0.88,1.34) | 0.441 |
| RP | *NA* | | 1.09(0.82,1.44) | 0.567 |
| NB | *NA* | | 1.19(0.90,1.57) | 0.235 |
| ZIP | 2.56(0.95,6.56) | **0.050** | 0.92(0.71,1.19) | 0.537 |
| ZIRP | 2.56(0.9,8.07) | 0.108 | 0.92(0.66,1.28) | 0.628 |
| ZINB^$^ | 6.56(0.95,32.26) | **0.021** | 0.97(0.71,1.32) | 0.839 |
| ZIGP^$^ | 8.04(0.89,45.11) | **0.018** | 0.87(0.63,1.21) | 0.411 |
| ZHP | 1.64(1.01,2.67) | **0.045** | 0.94(0.69,1.29) | 0.714 |
| ZHNB | 1.64(1.01,2.67) | **0.045** | 0.98(0.7,1.36) | 0.886 |
| **Example dataset 3: After the GI fellowship program vs. before the GI fellowship program*** | | | | |
| LR | 1.64(1.36,1.97) | **<0.001** |  | |
| PR | *NA* | | 1.61(1.46,1.78) | **<0.001** |
| RP | *NA* | | 1.61(1.39,1.87) | **<0.001** |
| NB | *NA* | | 1.60(1.39,1.85) | **<0.001** |
| ZIP | 1.38(0.97,1.83) | 0.027 | 1.39(1.21,1.60) | **<0.001** |
| ZIRP | 1.38(0.95,1.89) | 0.049 | 1.39(1.15,1.68) | **0.001** |
| ZINB** | 1.83(0.64,8.67) | 0.446 | 1.57(1.34,1.84) | **<0.001** |
| ZIGP** | 1.35(0.23,7.75) | 0.499 | 1.49(1.28,1.75) | **<0.001** |
| ZHP | 1.64(1.36,1.97) | **<0.001** | 1.40(1.22,1.61) | **<0.001** |
| ZHNB | 1.64(1.36,1.97) | **<0.001** | 1.54(1.23,1.94) | **<0.001** |

OR: Odds Ratio; RR: Risk Ratio; CI: Confidence Interval; NA: Not Applicable; LR: Logistic Regression; PR: Poisson Regression; RP: Robust Poisson; NB: Negative Binomial; ZIP: Zero-inflated Poisson; ZIRP: Zero-inflated Robust Poisson; ZINB: Zero-inflated Negative Binomial; ZIGP: ZIGP: Zero-inflated Generalized Poisson; ZHP: Zero hurdle Poisson; ZHNB: Zero hurdle Negative Binomial; CAC: Cap-Assisted Colonoscopy; SC: Standard Colonoscopy; GI: Gastroenterology; ^#^ Adjusted for total procedure time ^$^ inflated part of the model was only adjusted for age, sex, and total procedure time; *Adjusted for age, body mass index, sex, time of day, and sedation type. **Inflated part of the models only adjusted for age, sex, and body mass index; Highlighted p-values are significant findings.

Supplementary Table 4. Comparison of polyp detection rate and the number of detected polyps between the presence and absence of the GI fellowship program after imputing missing data (N=2127)

| **Models** | **Polyp detection rate** | | **Number of detected polyps** | |
| --- | --- | --- | --- | --- |
|  | **OR (95% CI)** | **p-value** | **RR (95% CI)** | **p-value** |
| **Example dataset 3: After the GI fellowship program vs. before the GI fellowship program*** | | | | |
| LR | 1.65(1.38,1.97) | **<0.001** | *NA* | |
| PR | *NA* | | 1.67(1.52,1.84) | **<0.001** |
| RP | *NA* | | 1.67(1.45,1.92) | **<0.001** |
| NB | *NA* | | 1.66(1.45,1.90) | **<0.001** |
| ZIP | 1.27(0.92,1.68) | 0.088 | 1.50(1.31,1.71) | **<0.001** |
| ZIRP | 1.27(0.88,1.75) | 0.131 | 1.50(1.24,1.80) | **<0.001** |
| ZINB^$^ | 3.02(0.63,55.46) | 0.456 | 1.62(1.40,1.87) | **<0.001** |
| ZIGP^#^ | 3.01(0.71,30.08) | 0.349 | 1.49(1.31,1.71) | **<0.001** |
| ZHP | 1.65(1.38,1.97) | **<0.001** | 1.50(1.25,1.81) | **<0.001** |
| ZHNB | 1.65(1.38,1.97) | **<0.001** | 1.67(1.33,2.09) | **<0.001** |

OR: Odds Ratio; RR: Risk Ratio; CI: Confidence Interval; NA: Not Applicable; LR: Logistic Regression; PR: Poisson Regression; RP: Robust Poisson; NB: Negative Binomial; ZIP: Zero-inflated Poisson; ZIRP: Zero-inflated Robust Poisson; ZINB: Zero-inflated Negative Binomial; ZIGP: ZIGP: Zero-inflated Generalized Poisson; ZHP: Zero hurdle Poisson; ZHNB: Zero hurdle Negative Binomial; *Adjusted for age, sex, body mass index, sex, time of day, and sedation type; ^$^ inflated part of the model was only adjusted for age, sex, and body mass index; ^#^inflated part of the model was only adjusted for age, sex, and body mass index; Highlighted p-values are significant findings.

Supplementary Table 5. Associated factors with polyp detection rate and the number of detected polyps in example data 3

| **Models** | **LR** | | **ZIRP** | | **ZINB** | |
| --- | --- | --- | --- | --- | --- | --- |
| **Polyp detection** | **OR (95% CI)** | **p-value** | **OR (95% CI)** | **p-value** | **OR (95% CI)** | **p-value** |
| Fellowship-presence | 1.64 (1.36, 1.97) | <0.001 | 1.41 (1.94, 1.02) | 0.039 | 2.25(0.64,7.85) | 0.208 |
| Age (years) | 1.02 (1.01, 1.04) | 0.001 | 1.02 (1.05, 1.00) | 0.098 | 1.25(0.57,2.72) | 0.593 |
| Body mass index (kg/m^2^) | 1.02 (1.01, 1.04) | 0.003 | 1.02 (1.06, 0.98) | 0.259 | 1.21(0.98,1.48) | 0.077 |
| Sex -male | 1.55 (1.28, 1.89) | <0.001 | 1.27 (1.74, 0.93) | 0.130 | 1.49(0.43,5.21) | 0.534 |
| Time of day-PM | 1.48 (1.21, 1.80) | <0.001 | 1.38 (1.96, 0.97) | 0.074 | 0.57(0.02,20.91) | 0.760 |
| Sedation-moderate | 0.75 (0.39, 1.44) | 0.395 | 1.05 (2.21, 0.49) | 0.907 | 1.86(0.12,29.96) | 0.663 |
|  | **PR** | |  |  |  |  |
| **Detected polyp counts** | **RR (95% CI)** | **p-value** | **RR (95% CI)** | **p-value** | **RR (95% CI)** | **p-value** |
| Fellowship-presence | 1.57 (1.42, 1.74) | <0.001 | 1.35 (1.14, 1.61) | 0.001 | 1.49(1.17,1.90) | 0.001 |
| Age (years) | 1.02 (1.01, 1.03) | <0.001 | 1.01 (1.00, 1.02) | 0.081 | 1.01(0.99,1.02) | 0.239 |
| Body mass index (kg/m^2^) | 1.02 (1.01, 1.03) | <0.001 | 1.01 (0.99, 1.03) | 0.260 | 1.01(0.98,1.04) | 0.453 |
| Sex -male | 1.53 (1.38, 1.69) | <0.001 | 1.39 (1.18, 1.63) | <0.001 | 1.49(1.24,1.79) | <0.001 |
| Time of day-PM | 1.38 (1.24, 1.55) | <0.001 | 1.20 (0.99, 1.46) | 0.069 | 1.42(1.17,1.72) | <0.001 |
| Sedation-moderate | 0.61 (0.47, 0.79) | <0.001 | 0.62 (0.44, 0.86) | 0.005 | 0.58(0.35,0.95) | 0.031 |

OR: Odds Ratio; RR: Risk Ratio; CI: Confidence Interval; LR: Logistic Regression; PR: Poisson Regression; ZIRP: Zero-inflated Robust Poisson; ZINB: Zero-inflated Negative Binomial.
