# Supplementary material for: Zero-inflated models for the evaluation of colorectal polyps in colon cancer screening studies—a value-based biostatistics practice
Source: PeerJ. 2025 May 26;13:e19504. doi: 10.7717/peerj.19504 (PMC12121622; doi:10.7717/peerj.19504)
Supplement: Supplemental Information 2 [file peerj-13-19504-s002.docx]

Dataset 1:

nid: patient unique identifier

number_of_polyps_1: number of polyps detected

study_arm: Cap-assisted colonoscopy vs. standard colonoscopy

total_procedure_time: procedure time in seconds

age: age in years

sex: 1: male; 2: female

Dataset 2:

nid: patient unique identifier

number_of_polyps_1: number of polyps detected

segmental: Segmental vs. standard colonoscopy

total_procedure_time: procedure time in minutes

age: age in years

sex_c: male and female

Dataset 3:

nid: patient unique identifier

number_of_polyps_1: number of polyps detected

fellowship: before and after GI fellowship introduction

gender_m: 1: male; 0: female

age_at_colonoscopy: age in years

time_of_the_day_am: toiming of procedure (am or pm)

bmi: body mass index (kg/m^2^)

sedation_moderate: moderate vs. others
